# Partially Ablative Body Radiation Therapy Versus Standard Palliative Radiation Therapy for Locally Advanced Bulky Unresectable Sarcomas

**DOI:** 10.1016/j.adro.2026.102070

**Published:** 2026-04-30

**Authors:** Christopher Williams, Julie Chu, SuChen Fong, David Chang, TaeWoo Im, Adam Yeo, David Jong, Mark Burns, Siena Williams, Therese Chesson, Tim Spelman, Bianca de Dios, Sarat Chander

**Affiliations:** a Faculty of Medicine, The University of Queensland, St Lucia, Australia; b Department of Radiation Oncology, Royal Brisbane and Women’s Hospital, Brisbane, Australia; c Department of Radiation Oncology, Peter MacCallum Cancer Centre, Melbourne, Australia; d Sir Peter MacCallum Department of Oncology, University of Melbourne, Melbourne, Australia; e Department of Health Services Research, Peter MacCallum Cancer Centre, Melbourne, Australia

## Abstract

**Purpose:**

Locally advanced soft tissue sarcomas cause significant tumor mass effects, often leading to onerous symptoms. Our institution has previously developed partially ablative body radiation therapy (PABR) for palliation in these circumstances, which has demonstrated effective symptom relief. However, these outcomes have not been compared with clinical outcomes from standard palliative radiation therapy (SPR).

**Methods and Materials:**

A total of 57 patients (30 PABR and 27 SPR) with various soft tissue sarcomas >5 cm in diameter treated between December 2019 and April 2024 were retrospectively reviewed. The primary endpoints were symptomatic and objective structural response rates and were compared using binomial regression. Secondary endpoints were overall survival, freedom from local progression, and freedom from distant progression.

**Results:**

The median age was 73 years and 56 years in the PABR and SPR groups, respectively. The median tumor volume was 898.55 cm^3^ and 205 cm^3^, respectively. The predominant dose and fractionation were 20 Gy in 5 fractions with an intratumoral boost to 50 Gy (90.0%) in the PABR group, while 30 Gy in 10 fractions (48.1%) was most common in the SPR group. After a median follow-up of 9 months, 90% of patients in the PABR group experienced symptomatic improvement compared with 70% in the SPR group. In multivariate analysis, PABR was associated with significantly higher rates of symptomatic improvement (adjusted relative risk, 1.30; 95% CI, 1.03-1.63; *P* = .026). Regarding objective response, 73.3% versus 48.2% of patients exhibited a partial response, with stable disease observed in 13.3% versus 11.1% of patients in the PABR and SPR groups, respectively. Mean absolute tumor volume reduction was 38% versus 35%. One-year estimated overall survival was 57.4% (95% CI, 35.6%-74.2%) versus 40.7% (95% CI, 22.5%-58.2%), freedom from local progression was 69.7% (95% CI, 43.2%-85.6%) versus 33.1% (95% CI, 15.0%-52.6%), and freedom from distant progression was 46.8% (95% CI, 23.4%-67.2%) versus 3.7% (95% CI, 0.3%-15.9%) for the PABR and SPR groups, respectively.

**Conclusions:**

PABR for bulky unresectable sarcomas may provide improved palliation of symptoms compared to SPR, with potentially better local control despite treating significantly larger tumors.

## Introduction

Soft tissue sarcomas (STS) are a rare and heterogeneous group of tumors. Locally advanced, bulky sarcomas are often unresectable and cause significant tumor mass effects, leading to onerous symptoms. Current treatment options to effectively palliate locally advanced sarcomas are limited due to several inherent challenges.

Surgical resection of locally advanced STS is technically challenging and sometimes unachievable because of proximity to vital structures, particularly in retroperitoneal sarcomas.^1^ STS are also known to be chemo-resistant, with common high-grade STS demonstrating estimated response rates of only 20% to 28% to chemotherapy, while also carrying significant toxicity profiles.^2^, ^3^, ^4^ Response to conventional radiation therapy (RT) is similarly limited because of the radioresistant nature of sarcomas and the inability to deliver high doses without increased toxicity to adjacent structures.^5^^,^^6^ These factors highlight the need to develop more effective palliative approaches for unresectable sarcomas.

Recent data have demonstrated improved local control in sarcoma metastases with the delivery of high biologically equivalent doses (BEDs) via an stereotactic ablative radiation therapy approach.^7^ A similar approach that may be applied to both metastatic and primary sarcomas involves delivering a high “ablative” dose to the central portion of the tumor while accepting lower peripheral doses to spare organs-at-risk (OARs). Preliminary studies of this technique have demonstrated promising results, showing reasonable local control rates with acceptable safety profiles.^8^^,^^9^ Building on this concept, our institution has developed a protocol termed partially ablative body radiation therapy (PABR).

Previous work from our institution has demonstrated encouraging outcomes with PABR in patients with bulky, unresectable sarcomas, including favorable symptomatic response, tumor volume reduction, and acceptable toxicity profiles.^10^ However, these results have not been directly compared with outcomes from standard palliative radiation therapy (SPR) approaches. This study aims to report the comparative clinical outcomes of PABR versus SPR in patients with bulky, unresectable sarcomas, with a focus on symptom palliation efficacy.

## Methods and Materials

### Study design and patient selection

**Figure 1:**
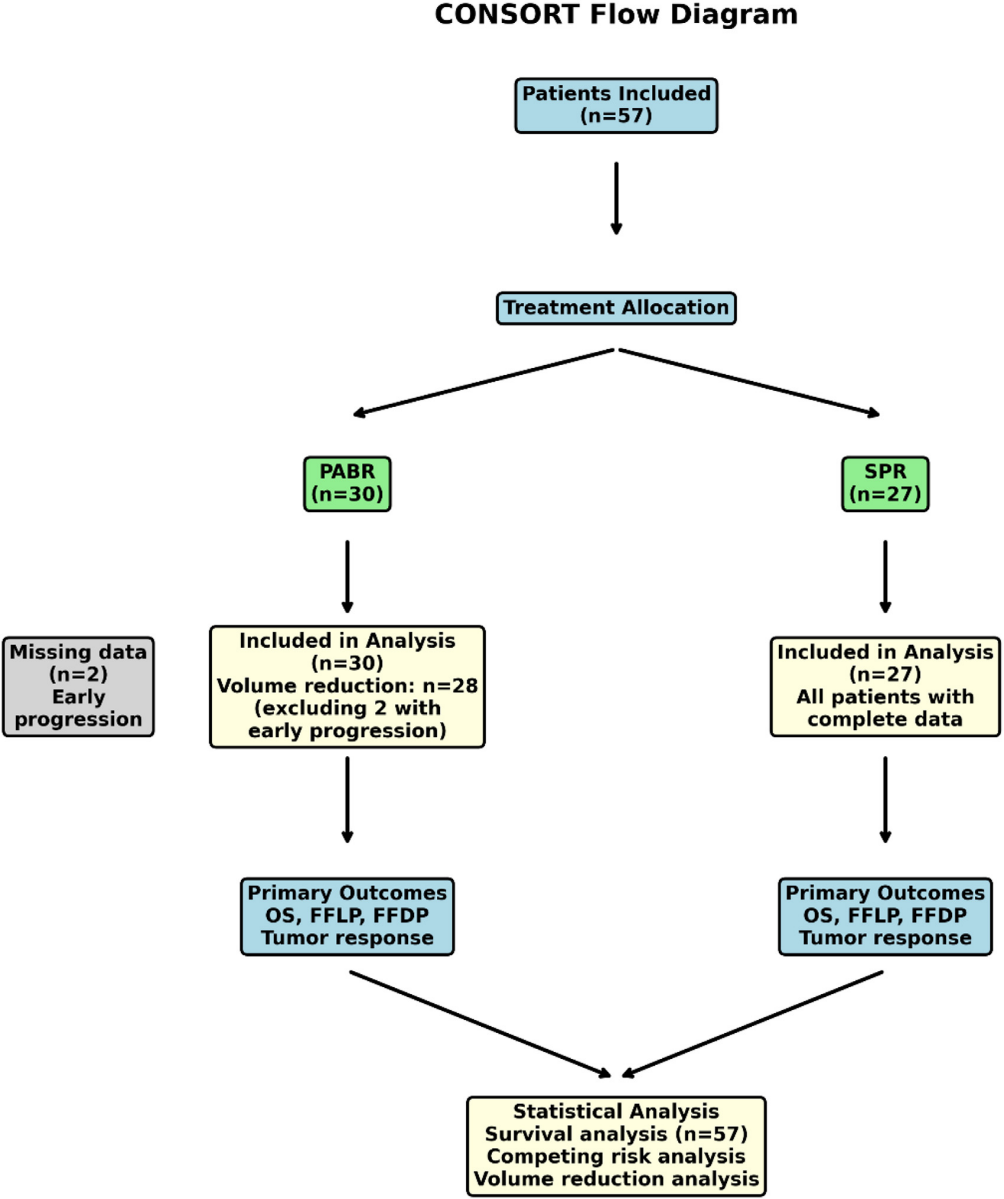
Consort diagram of patient inclusion. *Abbreviations:* FFDP = freedom from distant progression; FFLP = freedom from local progression; OS = overall survival; PABR = partially ablative body radiation therapy; SPR = standard palliative radiation therapy.

This retrospective study was conducted with approval from the local institutional research ethics committee. The study population comprised adults with locally advanced unresectable sarcomas treated with either PABR or SPR. Inclusion criteria consisted of the following: histologically confirmed sarcoma reviewed by a sarcoma pathologist, any stage of disease, minimum tumor diameter of 5 cm, and cases deemed unresectable after multidisciplinary discussion or refusal of other conventional treatment options. All patients were treated between December 2019 and April 2024 at a single center (Fig. 1).

### PABR technique

The PABR protocol delivers a palliative dose to the peripheral gross tumor volume (GTV) while simultaneously delivering a higher integrated boost dose to the central portion of the GTV (GTVboost). Treatment is administered 2 to 3 times weekly on nonconsecutive days using volumetric modulated arc therapy (VMAT).

For target delineation, diagnostic imaging (computed tomography [CT]/magnetic resonance imaging and/or positron emission tomography–CT) was fused with the planning scan to contour the GTV. A 1.5 cm isotropic contraction from the GTV (ranging from 1 to 2 cm depending on tumor size and proximity to critical organs) was applied to form the GTVboost within the intact GTV. This distance allows sufficient space for dose falloff between the 2 different dose levels (1.5-3 Gy falloff/mm). A 1.0 cm isotropic expansion from the GTV generated the planning target volume (PTV).

Planning goals were defined as follows: D95% coverage >95% of the prescription dose (20 Gy) for PTV, D95% >prescribed dose (20 Gy) for the GTV, and >D90% prescribed boost dose (50 Gy) for the GTVboost structure. The hotspot (D2%) was maintained within 130% of the prescribed boost dose (with acceptable variation). VMAT optimization prioritized (1) accurate D95% coverage of the PTV without dose spillage to surrounding OARs and (2) D2% hotspot centered within the GTVboost to ensure robust PABR delivery and to allow for any anatomic changes and setup uncertainties.

Patient immobilization and image guided RT delivery followed institutional protocols, including daily cone beam CT matching around the intact GTV using predefined image guided RT structures.

Please refer to the previous publication by Jong et al^11^ in *Practical Radiation Oncology* for further details on the PABR technique.

### SPR technique

Patients in the SPR group received conventional palliative RT using 3-dimensional conformal or intensity modulated RT/VMAT techniques based on institutional protocols. Common dose and fractionation schedules included: 30 Gy in 10 fractions, 20 Gy in 5 fractions, and 36 Gy in 12 fractions, all delivered in 5 fractions per week. Treatment planning and prescriptions adhered to International Commission on Radiation Units and Measurments standards.

### Follow-up and assessment

Demographic, clinicopathological, treatment, and toxicity data were prospectively collected. Patients were followed with clinical examination and imaging (CT and magnetic resonance imaging) at the discretion of the treating physician (typically 3-month intervals). All but 2 patients had at least one set of posttreatment imaging (due to deaths prior to standard surveillance).

The primary assessment of symptomatic response involved physician-reported improvement in symptoms compared to baseline, evaluated at each follow-up interval (typically at 6-8 weeks posttreatment, then at 3-month intervals).

For objective structural response evaluation, clinical imaging was assessed using the Response Evaluation Criteria in Solid Tumors version 1.1. Additionally, absolute volumes of target tumors at baseline and posttreatment were measured and compared. To obtain the absolute volume measurements, images were imported into the treatment planning system, GTVs were contoured, and volumes calculated in cubic centimeters.

Secondary outcomes included overall survival (OS), freedom from local progression (FFLP), freedom from distant progression (FFDP), and safety analysis. OS was defined as the duration from RT initiation to the date of last follow-up or death. FFLP was defined as the time from RT commencement to the date of last follow-up or radiologic detection of local progression of the treated lesion. FFDP was defined as the time from RT commencement to the date of last follow-up or radiologic detection of new distant progression.

Toxicity was assessed according to the National Cancer Institute Common Terminology Criteria for Adverse Events version 5. Acute toxicity included events occurring within 90 days from treatment initiation, while late toxicity encompassed events occurring beyond 90 days.

### Statistical analysis

Continuous variables were summarized using mean and SD or median and IQR, as appropriate. Categorical variables were reported as frequencies and percentages. Baseline characteristics of patients and treatment outcomes were compared between PABR and SPR groups using a χ^2^ test, Fisher’s exact test, *t* test, or rank-sum test as indicated. The Kaplan-Meier method was employed to estimate and visualize time-to-event outcomes (OS, FFLP, and FFDP). Survival estimates were reported at 1 year as point estimates with associated 95% CI. A Fine-Gray analysis was also used to assess FFLP and FFDP to account for death as a competing risk. Comparisons between PABR and SPR groups used the log-rank test, with statistical significance set at *P* <.05. Analysis was performed using Stata version 18 (StataCorp. 2023; Stata Statistical Software: Release 18; StataCorp LLC).

## Results

### Patient characteristics

A total of 57 patients with bulky (≥5 cm) unresectable sarcomas were included: 30 in the PABR group and 27 in the SPR group. Baseline characteristics showed heterogeneity between the treatment cohorts (Table 1). The median age was 73 years (IQR, 68-77 years) in the PABR group compared to 56 years (IQR, 35-72 years) in the SPR group (*P* = .001). The median follow-up was 7.5 months (IQR, 4-14 months) in the PABR group and 10 months (IQR, 5-16 months) in the SPR group. The PABR group had a higher proportion of patients with reduced performance status (Eastern Cooperative Oncology Group, 2-3) compared with the SPR group.

**Table 1:** Baseline characteristics of patients

Characteristic	PABR (n = 30)	SPR (n = 27)	*P* value
Sex, n (%)			
Male	21 (70.0%)	17 (63.0%)	.574
Female	9 (30.0%)	10 (37.0%)	
Age, y			
Median (IQR)	73 (68, 77)	56 (35, 72)	.001
ECOG performance status, n (%)			.042
0-1	16 (59.3%)	22 (81.5%)	
2-3	11 (40.7%)	5 (18.5%)	
AJCC 8th edition stage, n (%)			<.001
II	5 (18.5%)	0 (0.0%)	
III (IIIB)	17 (63.0%) (47.0%)	2 (7.4%)	
IV	5 (18.5%)	25 (92.6%)	
Histologic subtype, n (%)			.189
Dedifferentiated liposarcoma	8 (29.6%)	6 (22.2%)	
Leiomyosarcoma	7 (25.9%)	5 (18.5%)	
Myxoid liposarcoma	3 (11.1%)	4 (14.8%)	
Other	9 (33.3%)	12 (44.4%)	
Anatomic site treated, n (%)			.368
Retroperitoneum	15 (55.6%)	12 (44.4%)	
Extremity	5 (18.5%)	8 (29.6%)	
Trunk	4 (14.8%)	3 (11.1%)	
Other	3 (11.1%)	4 (14.8%)	
Initial tumor volume, cm^3^			
Median (IQR)	898.55 (668.80, 2556.30)	205.00 (132.90, 577.20)	<.001
Mean (SD)	1986.73 (2698.12)	471.94 (535.33)	<.001
Previous systemic therapy, n (%)			.021
Yes	8 (29.6%)	6 (22.2%)	
No	19 (70.4%)	21 (77.7%)	
Dose and fractionation, n (%)			<.001
50 Gy/20 Gy/5 fx SIB	24 (90.0%)	0 (0.0%)	
30 Gy/25 Gy/5 fx SIB	1 (3.3%)	0 (0.0%)	
24 Gy/16 Gy/2 fx SIB	1 (3.3%)	0 (0.0%)	
20 Gy/2 fx, 25 Gy/10 fx sequential	1 (3.3%)	0 (0.0%)	
20 Gy/5 fx	0 (0.0%)	11 (40.1%)	
30 Gy/10 fx	0 (0.0%)	13 (48.1%)	
36 Gy/12 fx	0 (0.0%)	1 (3.7%)	

*Abbreviations:* AJCC = American Joint Commitee on Cancer; ECOG = Eastern Cooperative Oncology Group; fx = fractions; PABR = partially ablative body radiation therapy; SIB = simultaneous integrated boost; SPR = standard palliative radiation therapy.

Disease characteristics differed between the groups, with the PABR arm having a higher proportion of patients with more locally advanced disease. The median tumor volume prior to treatment was 898.55 cm^3^ (IQR, 668.80-2556.30 cm^3^) in the PABR group versus 205.00 cm^3^ (IQR, 132.90-577.20 cm^3^) in the SPR group (*P* <.001). Disease staging also varied, with the PABR arm predominantly comprised of stage III disease (47% stage IIIB), whereas the majority of patients in the SPR arm had stage IV disease (92.6%) at treatment initiation.

Histologic subtypes varied between the groups. The study included multiple sarcoma subtypes, with dedifferentiated liposarcomas, leiomyosarcomas, and myxoid liposarcomas being the most frequent. Approximately half of all patients had tumors located in the retroperitoneum.

The predominant dose and fractionation schedule in the PABR group was 50 Gy/20 Gy in 5 fractions with simultaneous integrated boost (90.0%), while in the SPR group, 30 Gy in 10 fractions (48.1%) followed by 20 Gy in 5 fractions (40.1%) were most common. Estimated BED_4_ for PABR GTVboost was 175 Gy_4_ and for SPR GTV, 40 to 63 Gy_4_.

### Treatment response

#### Symptomatic response

At a median follow-up of 11.8 months, analysis of symptomatic patients demonstrated improvement in 90% (18 of 20) of patients in the PABR group compared to 70% (19 of 27) in the SPR group. After adjusting for age, performance status, stage of treated tumor, and TNM staging, PABR was associated with a significantly higher rate of symptomatic response compared with SPR (adjusted Relative Risk, 1.3; 95% CI, 1.03-1.63; *P* = .026).

#### Objective response

Objective response assessment revealed that 73.3% of patients in the PABR group exhibited a partial response compared with 48.2% in the SPR group (*P* = .008). Stable disease was observed in 13.3% and 11.1% of patients, respectively. When combining partial response and stable disease rates, the disease control rate was 86.6% in the PABR group versus 59.3% in the SPR group.

#### Volume reduction

**Figure 2:**
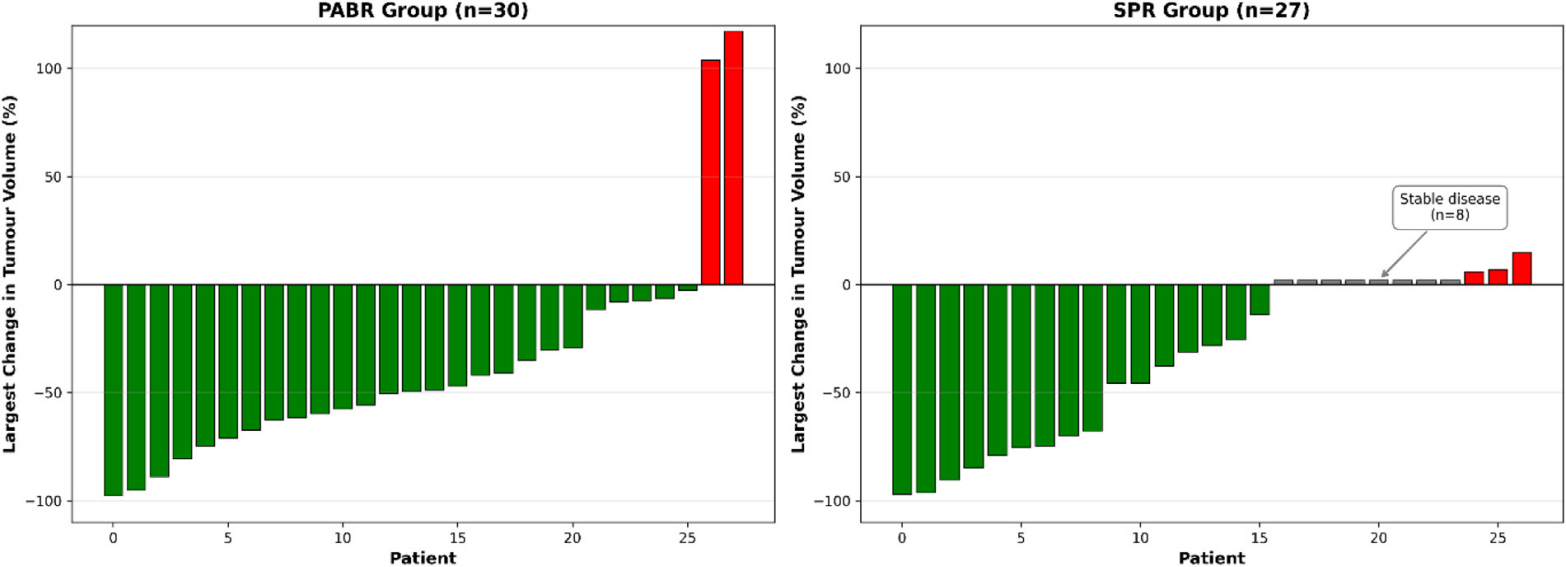
Waterfall plot of change of tumour volume for (*left*) PABR and (*right*) SPR.

Analysis of tumor volume changes in the overall cohort demonstrated a mean reduction of 38% in the PABR group versus 35% in the SPR group. However, an exploratory analysis excluding 2 patients from the PABR group, whose tumors doubled in volume in 3 months after treatment (with death occurring shortly thereafter), was performed to evaluate the impact of better patient selection. Among the remaining PABR patients, mean tumor volume reduction was 49% (compared with 35% in the SPR group), and the partial response rate increased to 78% (Fig. 2).

### Survival outcomes

**Figure 3:**
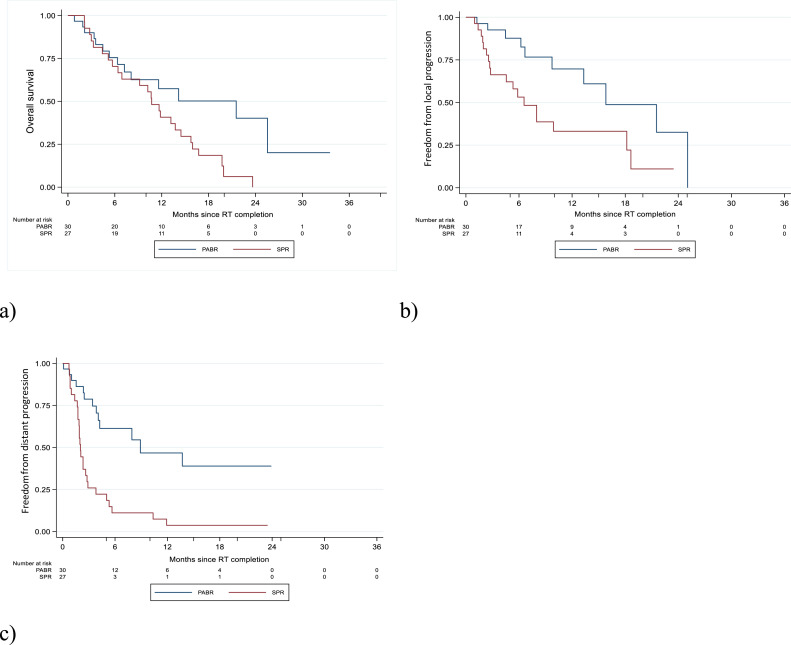
Kaplan-Meier curves of (a) overall survival, (b) freedom from local progression, and (c) freedom from distant progression for patients treated with partially ablative body radiation therapy versus standard palliative radiation therapy. *Abbreviations:* PABR = partially ablative body radiation therapy; RT = radiation therapy; SPR = standard palliative radiation therapy.

Kaplan-Meier estimates for 1-year OS, FFLP, and FFDP are presented in Fig. 3 and summarized in Table 2. One-year OS was 57.4% (95% CI, 35.6%-74.2%) in the PABR group versus 40.7% (95% CI, 22.5%-58.2%) in the SPR group. FFLP was 69.7% (95% CI, 43.2%-85.6%) in the PABR group compared with 33.1% (95% CI, 15.0%-52.6%) in the SPR group.

**Table 2:** Treatment outcomes

Outcome	PABR (n = 30)	SPR (n = 27)	*P* value
Symptomatic improvement, n (%)	18/20 (90%)*	19/27 (70%)	
Tumor response, n (%)			.008
Partial response	22 (73.3%)	13 (48.2%)	
Stable disease	4 (13.3%)	3 (11.1%)	
Progressive disease	2 (6.7%)	11 (40.7%)	
Disease control rate (partial response + stable disease), %	86.6%	59.3%	.022
Tumor volume change, %			
Mean volume reduction	38%	35%	.790
Mean volume reduction repeat analysis†	49%	35%	.042
Toxicity (CTCAE v5.0), n (%)			
Grade 1/2	19 (70.4%)	19 (70.4%)	-
Grade 3	1 (3.7%)	1 (3.7%)	-
Grade 4/5	0 (0.0%)	0 (0.0%)	-
One-year survival estimates, % (95% CI)			
Overall survival	57.4% (35.6%-74.2%)	40.7% (22.5%-58.2%)	.035
Freedom from local progression	69.7% (43.2%-85.6%)	33.1% (15.0%-52.6%)	.015
Freedom from distant progression	46.8% (23.4%-67.2%)	3.7% (CI, 0.3%-15.9%)	<.001

*Abbreviations:* CTCAE = National Cancer Institute Common Terminology Criteria for Adverse Events; PABR = partially ablative body radiation therapy; SPR = standard palliative radiation therapy.

⁎ Analysis excluding 10 patients who were asymptomatic at the time of treatment.

† Analysis excluding 2 patients whose tumors doubled in volume posttreatment in the PABR arm.

FFDP was 46.8% (95% CI, 23.4%-67.2%) at 1 year in the PABR group versus 3.7% (95% CI, 0.3%-15.9%) in the SPR group.

Given that the stage distribution differed between treatment groups, with the PABR cohort predominantly comprising stage III disease (47% stage IIIB) and the majority of patients in the SPR cohort having stage IV disease (92.6%), a competing risk analysis was performed for FFLP and FFDP to account for death as a competing event. Using the Fine-Gray model, the subdistribution hazard ratio for local progression was 0.44 (95% CI, 0.21-0.93; *P* = .031) and for distant progression was 0.24 (95% CI, 0.12-0.47; *P* < .001), both favoring PABR.

### Toxicity

A single grade 3 toxicity (colitis) and no late toxicity of any grade were observed in the PABR group. Acute grade 1 to 2 toxicities included nausea, fatigue, diarrhea, dermatitis, and pain flare, all of which resolved within 4 weeks of completing RT.

In the SPR group, a comparable toxicity profile was observed, with one grade 3 event (enteritis). Grade 1 to 2 toxicities were primarily fatigue, nausea, and skin reactions.

## Discussion

This study represents the first direct comparison of PABR versus SPR for bulky, unresectable sarcomas. Our findings demonstrate superior symptom control and objective response rates with PABR despite treating significantly larger tumors, without increased toxicity.

The advantages of PABR over conventional RT techniques include the ability to deliver ablative doses to tumor centers while maintaining acceptable doses to surrounding normal tissues. The PABR protocol’s abbreviated course—5 fractions delivered twice weekly aligns with contemporary hypofractionation paradigms that have demonstrated comparable or superior outcomes to protracted regimens in various malignancies.^12^^,^^13^ This compact schedule not only optimizes resource utilization but potentially enhances treatment efficacy through times biologically effective doses (175 Gy_4_ for PABR versus 40-63 Gy_4_ for SPR). This dose escalation aligns with contemporary studies demonstrating that higher BED_4_ values improve local control, specifically in sarcoma patients.^7^^,^^14^

Our primary endpoint—symptomatic improvement—revealed a clinically meaningful difference favoring PABR (90% vs 70%). This superior palliative efficacy aligns with previous investigations of dose-intensified regimens for STS management.^15^^,^^16^ The observed response rates exceed previously reported outcomes in hypofractionated palliative RT series, which demonstrated symptomatic improvement in 79% to 83% of patients.^14^^,^^16^^,^^17^ Importantly, PABR achieved these results in a cohort of patients with significantly larger tumor volume while maintaining a convenient outpatient treatment schedule accessible to patients with substantial symptom burden.

The difference in objective response rates (73.3% for PABR vs 48.2% for SPR, *P* = .008) suggests that higher ablative doses may overcome the intrinsic resistance typically associated with sarcomas. The improved FFLP in the PABR cohort (69.7% vs 33.1% at 1 year) further supports this hypothesis. These findings corroborate established dose-response relationships in treatment-resistant tumors, including improved local control with dose escalation in STS, even in the challenging context of large, high-grade lesions.^7^^,^^8^^,^^14^^,^^18^

Although survival parameters numerically appear better in the PABR group (1-year OS and FFDP), these outcomes are likely to be attributable to the intercohort heterogeneity in demographics, tumor volume, histologic profiles, and disease staging. It will require both prospective study and longer follow-up to determine whether the observed increase in local control results in changes in other survival measures.

The toxicity profile observed in both treatment groups demonstrates the safety of modern RT techniques. Historical series of unresectable sarcoma patients have reported treatment-related morbidity of 8% to 27%, with higher rates associated with doses exceeding 63 Gy equivalent dose in 2Gy fractions.^6^ Our findings of minimal grade 3 toxicity (3.7% in both arms) and absence of grade 4 to 5 events compare favorably with these precedents. In the PABR group, the isotropic contraction methodology used to generate the high-dose region effectively limited doses to surrounding OARs, contributing to the favorable toxicity profile despite the higher central doses. This is consistent with contemporary evidence that modern RT planning and delivery techniques can safely achieve dose escalation while respecting normal tissue constraints.^19^

Several limitations warrant acknowledgment. The retrospective design introduces potential selection bias, and the significant intercohort heterogeneity necessitates that all outcomes should be viewed as hypothesis-generating. The inclusion of 10 asymptomatic patients in the PABR cohort, necessitated by the retrospective design, represents a potential source of bias in symptomatic response rate calculations, while the relatively modest sample size also magnifies the impact of individual events on the overall analysis, as evidenced by the disproportionate effect of 2 PABR patients whose tumors rapidly doubled posttreatment and one patient with poor performance status who died before surveillance imaging. These observations underscore the critical importance of thorough patient selection for this RT technique.

Our findings emphasize that optimal PABR candidate selection is important. The improved response rates after excluding patients with rapid disease progression suggest that individuals with aggressive tumor biology may derive less benefit from this approach. Whether different histologies predict clinical course cannot be deduced from these data. Volume change from between diagnostic scan and simulation, or between simulation and start of treatment, may serve as better predictors of clinical course, although further data are needed to examine this hypothesis fully.

Patients with poor performance status who may not survive long enough to experience treatment benefits should also be carefully evaluated. For such patients, less resource-intensive RT approaches may offer comparable symptomatic improvement.^20^

Patient selection in the future would benefit from a comprehensive assessment of disease characteristics, performance status, and anticipated survival. Validated prognostic models for palliative RT that integrate multiple factors, including performance status, primary disease site, and metastatic burden, could potentially be adapted to guide appropriate selection of candidates for PABR.^21^

Additional methodological limitations include the relatively brief follow-up duration, limiting survival analysis. The absence of systematically collected quality-of-life metrics represents another constraint, as such measures would provide valuable insights into treatment impact. Contemporary palliative oncology research increasingly prioritizes patient-reported outcomes as essential endpoints, particularly in palliative settings where quality of life often supersedes traditional oncologic parameters in clinical relevance.^22^ This group has started collecting patient-reported outcomes as part of an ongoing prospective trial to address this limitation.

## Disclosures

Sarat Chander reports that financial support was provided by the Australia and New Zealand Sarcoma Association. The other authors declare that they have no known competing financial interests or personal relationships that could have appeared to influence the work reported in this paper.
